# Analysis of Evolution, Expression and Genetic Transformation of TCP Transcription Factors in Blueberry Reveal That *VcTCP18* Negatively Regulates the Release of Flower Bud Dormancy

**DOI:** 10.3389/fpls.2021.697609

**Published:** 2021-07-09

**Authors:** Yongqiang Li, Shuang An, Qiangqiang Cheng, Yu Zong, Wenrong Chen, Weidong Guo, Lu Zhang

**Affiliations:** ^1^Key Laboratory of Silviculture, Co-Innovation Center of Jiangxi Typical Trees Cultivation and Utilization, College of Forestry, Jiangxi Agricultural University, Nanchang, China; ^2^Zhejiang Provincial Key Laboratory of Biotechnology on Specialty Economic Plants, College of Chemistry and Life Sciences, Zhejiang Normal University, Jinhua, China

**Keywords:** blueberry, TCP transcription factors, expression profiles analysis, flower bud dormancy, transgenic plants

## Abstract

Plant-specific TEOSINTE BRANCHED 1, CYCLOIDEA, PROLIFERATING CELL FACTORS (TCP) transcription factors have versatile functions in plant growth, development and response to environmental stress. Despite blueberry’s value as an important fruit crop, the TCP gene family has not been systematically studied in this plant. The current study identified blueberry TCP genes (*VcTCPs*) using genomic data from the tetraploid blueberry variety ‘Draper’; a total of 62 genes were obtained. Using multiple sequence alignment, conserved motif, and gene structure analyses, family members were divided into two subfamilies, of which class II was further divided into two subclasses, CIN and TB1. Synteny analysis showed that genome-wide or segment-based replication played an important role in the expansion of the blueberry TCP gene family. The expression patterns of *VcTCP* genes during fruit development, flower bud dormancy release, hormone treatment, and tissue-specific expression were analyzed using RNA-seq and qRT-PCR. The results showed that the TB1 subclass members exhibited a certain level of expression in the shoot, leaf, and bud; these genes were not expressed during fruit development, but transcript levels decreased uniformly during the release of flower bud dormancy by low-temperature accumulation. The further transgenic experiments showed the overexpression of *VcTCP18* in *Arabidopsis* significantly decreased the seed germination rate in contrast to the wild type. The bud dormancy phenomena as late-flowering, fewer rosettes and main branches were also observed in transgenic plants. Overall, this study provides the first insight into the evolution, expression, and function of *VcTCP* genes, including the discovery that *VcTCP18* negatively regulated bud dormancy release in blueberry. The results will deepen our understanding of the function of *TCPs* in plant growth and development.

## Introduction

*TCP* genes are a plant-specific transcription factor family whose members play vital roles in plant growth and development by affecting cell proliferation and differentiation. The name of TCP transcription factor was based on the first four identified members: TEOSINTE BRANCHED1 (TB1) from *Zea mays*, CYCLOIDEA (CYC) from *Antirrhinum majus*, and PROLIFERATING CELL FACTORS 1 and 2 (PCF1 and PCF2) from *Oryza sativa* (Luo et al., 1996; Doebley et al., 1997). Predictions of the secondary structure show that the TCP domain is similar to the eukaryotic transcription factor bHLH. However, the TCP basic region is long, and one of the conserved regions contains a non-canonical basic-helix-loop-helix structure, which results in different DNA binding characteristics. Therefore, the TCP family was defined as a new family of transcription factors (Cubas et al., 1999; Kosugi and Ohashi, 2002; Navaud et al., 2007).

The 24 TCPs in *Arabidopsis thaliana* can be divided into two subfamilies, Class I (PCF or TCP-P) and Class II (TCP-C), based on their different domains. The most significant difference between these classes is four additional amino acids in the TCP domain of class II. Class II TCP proteins also have a specific sequence, namely, the R structure, which is rich in 18–20 arginine (Asp) (Cubas et al., 1999). Type II TCPs are further divided into two subcategories, CINCINNATA (CIN)-like TCP (CIN-TCP) and CYCLOIDEA/TEOSINTE BRANCHED1 (CYC/TB1) (Navaud et al., 2007). The former subcategory contains eight members (*AtTCP2/3/4/5/10/13/17/24*), five of which are regulated by miRNA319 (Palatnik et al., 2003).

Class I members in the TCP transcription factor family promote cell proliferation and growth, but class II members inhibit these processes (Herve et al., 2009; Li et al., 2019). Genes of the two classes play versatile functions in leaf type control, axillary bud meristem development, plant height development, asymmetry of floral organs, hormone signal transduction, and the response to biotic and abiotic stresses (Martin-Trillo and Cubas, 2010; Daviere et al., 2014; Kim et al., 2014; Nicolas and Cubas, 2016; Viola et al., 2016). *TCP* genes are also involved in the regulation of plant circadian rhythm. For example, TCP proteins regulate the circadian clock by binding to the TGGGC (C/T) element in *Arabidopsis thaliana* (Giraud et al., 2010). There are few studies on the functional information of Class I TCP members, which are involved in plant development, growth, and proliferation. For example, *AtTCP14* and *AtTCP15* regulate embryonic growth *via* the gibberellin signaling pathway during seed germination (Resentini et al., 2015), and *AtTCP15* directly acts on *GA20ox1*, *HBI1*, and *PRE6* to promote petiole growth and hypocotyl elongation (Ferrero et al., 2019). In *Arabidopsis*, a tcp23-knockout line has an early-flowering phenotype, while overexpression lines exhibit the reverse phenotype, which indicates that *AtTCP23* participates in the regulation of flowering (Balsemao-Pires et al., 2013). *AtTCP16* shows higher expression in developing microspores; 50% of aborted pollen was produced in *TCP16* RNA interference (RNAi) plants, which indicates that *AtTCP16* plays a crucial role in the early stages of pollen grain development (Takeda et al., 2006). Compared with class I members, the functions of most class II *TCP* genes have already been elucidated. Experimental evidence supports that the proteins may be involved in various plant life activities, such as axillary bud meristem development, leaf and flower development, branching, hormone signaling, and plant defense (Luo et al., 1996; Doebley et al., 1997; Aguilar-Martínez et al., 2007; Nicolas and Cubas, 2016). Previous research demonstrated that *AtTCP18* and *AtTCP12* were similar to *tb1* in protein sequence, expression pattern, and mutant phenotype; these genes were named *BRC1* and *BRC2*, respectively. Compared with wild-type plants, *BRC1*-mutant plants had more axillary bud germination, which demonstrated that *BRC* genes plays a negative regulatory role during germination and inhibits the elongation of axillary bud branches (Aguilar-Martínez et al., 2007). Environmental factors, such as light and plant density, also regulate *BRC1* gene expression. The evidence demonstrated that the type II TCP transcription factor, together with the FT-FD complex, controls the initiation of inflorescence in *Arabidopsis*, and TF directly binds to the *AP1* promoter to enhance its transcription and regulate flowering (Li et al., 2019). The *BRC1* homologous gene in cucumber inhibits lateral bud outgrowth *via* the direct suppression of *PIN3* functioning and auxin accumulation in axillary buds (Shen et al., 2019).

Many TCP family members have been characterized in other plants, such as grape (Jiu et al., 2019; Leng et al., 2019), tobacco (Chen et al., 2016), Moso Bamboo (Liu et al., 2018), wheat (Zhao et al., 2018), corn (Ding et al., 2019), upland cotton (Li et al., 2017), plum (Zhou et al., 2016), tea (Zhou et al., 2019), and strawberry (Wei et al., 2016). Blueberries (*Vaccinium* spp.) are known as the “king of berries,” with high nutritional and great commercial value. Despite the significant roles of *TCP* family genes in plant growth and development, few detailed analyses of these genes in blueberry are available. Dormancy is the stage that perennial woody plants must go through for the next seasonal cycles, and it is also the adaptive evolution of plants to survive the harsh winter. Previous studies identified the transcription factor *SHORT VEGETATIVE PHASE-LIKE* (*SVL*) in poplar, closely related to *Arabidopsis SVP*, and its downstream target was determined to be *TCP18* by chromatin immunoprecipitation (ChIP) experiments from transgenic plants. *SVL* and *TCP18* can be regulated by low temperature, which play a negative regulatory role in bud germination (Singh et al., 2018).

The present study analyzed blueberry *TCP* genes using the genomic data of the tetraploid blueberry variety ‘Draper’ and identified a total of 62 *TCP* genes. Comprehensive analyses were performed, including multisequence alignment, conserved motif, gene structure, *cis-*acting elements and syntenic analysis. The expression patterns of *VcTCPs* in diverse tissues, stages of fruit development, flower bud dormancy release, and in response to hormone treatment were analyzed using multiple transcriptome data. The expression patterns of TCP gene family members during the process of dormancy release in flower buds were verified using qRT-PCR. The further transgenic experiments showed the overexpression of *VcTCP18* significantly decreased the seed germination rate in contrast to the wild type. The bud dormancy phenomena as late-flowering, fewer rosettes and main branches were also observed in transgenic plants. Overall, this study provides the first insight into the evolution, expression, and function of *VcTCP* genes, including the discovery that *VcTCP18* negatively regulates bud dormancy release in blueberry. The results will broaden our understanding of the function of *TCPs* in plant growth and development.

## Materials and Methods

Plant materials were taken from the blueberry orchard of the Zhejiang key laboratory of biotechnology onspecialty economic plants in 2019. The variety is South highbush ‘O'Neal’. Shoots (spring shoots), leaves (tender leaf), flower buds (endo-dormancy flower buds), flowers (flower blooms) and fruits (‘O'Neal’ S4 fruit) (Yang et al., 2018) were immediately placed in liquid nitrogen and stored at −80°C for future use.

Artificial cold accumulation: On November 19, 2018, 1-year-old shoots (the flower buds in endodormancy stage) of ‘O'Neal’ trees were collected, incubated at 15°C for 3 days, and transferred to 4°C. The following culture conditions were used: day/night temperature 4°C; 16/8 h day/night; light intensity of 320 μmol ⋅ m^–2^ ⋅ s^–1^, and relative air humidity of 75%. The water was changed every 2 days, and the base of the branches was cut off to reveal new stubble. Low-temperature treatment was applied for 28 days, and 30 flower buds were collected every 4 days and frozen in liquid nitrogen. Samples were stored at −80°C for later use. Other branches were exposed to forcing conditions for bud break measurements. The end of endodormancy is considered to have been reached when the percentage budbreak is 50% (Yooyongwech et al., 2009). Three biological replicates were performed for all of the above treatments.

*Arabidopsis* (Col-0) and the transgenic plants seeds were cultivated in 1/2 MS medium for 1 week then transferred to soil and grown in the incubators (16 h photoperiods, 22°C and 70% relative humidity).

### Identification of Putative *VcTCP* Genes in Blueberry

Two different methods were used to identify *TCP* genes in the blueberry genome. First, the hidden Markov model (HMM) profile of the conserved TCP domain (PF03634) was downloaded from the Pfam database^1^, and HMMER software was used against a local blueberry protein sequence database. The E value was set to 0.01. Second, we used all of the *Arabidopsis* TCP protein sequences downloaded from the *Arabidopsis* Information Resource (TAIR) database^2^ as reference sequences, and a local BLAST comparison was used to screen the blueberry genome database. All VcTCP protein sequences were verified using the SMART database^3^. We used MEGA to remove repeated sequences, and the obtained non-redundant VcTCP protein sequences were further analyzed. The molecular weights (MWs), isoelectric points (pIs), and the grand average of hydrophilicity (GRAVY) of VcTCP proteins were analyzed using the ExPASy website^4^. The subcellular localizations of VcTCP proteins were predicted using WoLF PSORT^5^ (Leng et al., 2019).

### Multiple Sequence Alignment and Phylogenetic Analysis

Twenty-four *Arabidopsis* TCP protein sequences were retrieved from TAIR^2^, and poplar, rice and kiwifruit TCP protein sequences were downloaded from the Plant Transcription Factor Database^6^. *Antirrhinum* CYC and maize TB1 sequences were retrieved from the NCBI database^7^. MEGAX software was used to perform multisequence alignment of the full amino acid sequences of the TCP proteins of blueberry, *Arabidopsis*, rice, kiwifruit, *Antirrhinum* and maize. The program and operating parameters for the construction of an evolutionary tree were the neighbor-joining method with 1,000 bootstrap replicates using MEGAX. The replacement model was JTT + G + F.

### Conserved Motifs and Gene Structure Analysis

The online tool MEME^8^ was used to identify and analyze the conserved motifs of TCP proteins (parameter setting: maximum number of motifs: 5; maximum motif width: 60) (Bailey and Elkan, 1994). The CDS and corresponding genomic DNA sequences of *VcTCP* genes were acquired from the blueberry genome. GSDS^9^ was used to display the exon-intron organization *via* comparison of the coding sequences with their corresponding genomic sequences (Hu et al., 2015).

### Putative Promoter *Cis-*Acting Element Analysis

The 1,500 bp upstream of the coding regions of the *VcTCP* genes were extracted from the blueberry genome data. PlantCARE^10^ was used to search for putative *cis-*acting elements (Lescot et al., 2002).

### Chromosomal Location and Synteny Analysis

Based on the blueberry genome information, 62 *VcTCP* genes were located on the chromosome. For the collinearity analysis, first, BlastP was performed to search for potential homologous gene pairs in the blueberry genome and between blueberry and *Arabidopsis* (E < 10^–5^, first five matches). Second, the homologous pairs were used as an input file for MCScanX to identify syntenic chains and types of duplication mechanisms (Tang et al., 2008; Wang et al., 2012). Circos and Tbtools software^11^ were used for visualization (Krzywinski et al., 2009; Chen et al., 2020).

### Expression Pattern of the 62 *VcTCP* Genes

The expression values of *VcTCPs* in numerous tissues, root salt stress treatment and leaf methyl jasmonate (MeJA) treatment were obtained from the RNA sequencing data reported previously (Colle et al., 2019), the variety is “Draper.” The transcriptome data of ‘O'Neal’ flower bud endo-dormancy and eco-dormancy release from our laboratory were used to analyze the expression profiles of *VcTCPs*. The RNA into 21 samples (7 bud development stage × 3 replicates), which were sequenced on an Illumina HiSeqTM 4000 after the library was qualified by Agilent 2100 Bioanalyzer and ABI Step One Plus real-time PCR System. Transcriptomic sequencing was conducted on the hypanthia and young fruits of ‘O'Neal’ and ‘Bluerain’ during anthesis and the early developmental period, sequenced on an Illumina HiSeq-Xten platform, which were acquired from our laboratory were also used to analyze. Zero to one was used to calculate the expression values. The expression heatmap of *VcTCP* genes was generated using TBtools software (Chen et al., 2020).

### qRT-PCR Analysis

For reverse transcription, HiScript III RT SuperMix for qPCR (+gDNA wiper) (Nanjing Nuoweizan Biotechnology Co., Ltd.) was used to construct the cDNA library according to the instructions. The reverse-transcribed cDNA was used as a template for qRT-PCR. Quantitative primers were designed using the software Premier 5.0. The specificity of primers was determined by BLAST sequence comparison. The primers were synthesized by Hangzhou Qingke Biotechnology Co., Ltd. The primer sequences are shown in Table 1. The efficiency of the primers was tested using the standard curve method, and primers with efficiencies between 80% and 120% were used for subsequent experiments. The quantitative kit was 2 × SYBR Green qPCR Mix (High ROX) (Beijing Adelaide Biotechnology Co., Ltd.), which uses a two-step method to perform qPCR amplification reactions. Blueberry *GAPDH* was used as an internal reference gene, and data analyses were performed using the relative quantitative method (2^–ΔΔCt^). SPSS Statistics 21 software was used for significant difference analyses, and Prism was used for drawing.

**TABLE 1:** The primers used for qRT-PCR in the study.

Gene name	Forward primer(5′–3′)	Reverse primer(3′–5′)	Amplicon size
*VcTCP18*	ATCGAGGGATAAAGCAAGAGC	TCACGGCTTCCAGAGTTTG	86
*VcTCP5*	AGTCAAGGGAAAAGGCAAGAG	TGGGCAGATTGGGTTCATTG	90
*VcTCP21*	GCGGTCTGAAAATCGTGTAAAAG	CTCTTTCTCCTTCTCTTTCTCCG	73
*VcTCP55*	CGATAAACTCACCGAGCTACC	TCTGACTGTTGCCCCATAATG	102
*VcTCP26*	TTTAATGCCTACACCCGTCG	TCAGTTGGAAGATTCTGGCC	140
*VcTCP54*	CTTGGTAAGAGACTCCGTTCAG	CTCCAAACCTGCCCAAAATC	146
*VcTCP19*	CTACCCCTGTTGCCCTATTG	TTCACTTATACCACCGCCAC	128
*VcTCP60*	CCAAACCCGCACAAATCAAG	CTCGGCCTTCTACTTTAGTGTG	129

### Gene Cloning, Plant Transformation, and Screening of Transgenic Lines

Full-length *VcTCP18* cDNA was amplified using cDNA prepared from mRNA extracted from blueberry ‘O'Neal’ flower bud as templates. The primer sequences are shown in Table 2. *VcTCP18* cDNA were cloned into the pMD 19-T vector and transferred into the *E. coli* DH5α, verified with PCR, finally transferred into the vector pCAMBIA2300-3 × flag, *via XbaI, Hind* III double digestion and recovered with T4 DNA ligase. The recombinant plasmid pCAMBIA2300-CaMV35S*-VcTCP18*-3 × flag-CaMV poly(A) signal, which was subsequently transferred into *Agrobacterium* strain GV3101. The *Arabidopsis* transformation was conducted by floral dipping method. T0 seeds were screened by kanamycin, and T1 plants were confirmed by RT-PCR analysis. T3 generation homozygous lines were used for all analyses.

**TABLE 2:** Primers for cloning *VcTCP18.*

Gene name	Forward primer(5′–3′)	Reverse primer(3′–5′)	Amplicon size
*VcTCP18*	GCTCTAGAGCATGGATACC AACACCAACCC	CCCAAGCTTGGGTTATGTCTGATC ACCACCACG	1,077

### Phenotypic Observation of Transgenic Plants

The seeds of WT and transgenic plants were cultivated in 1/2 MS medium with and without stratification (4°C treatment 2 days). The germination rate was counted every 6 h. Flowering time was measured by counting the number of days from sowing until flower buds were visible by the naked eye, meanwhile the number of rosette leaves were counted, main branches were also recorded (Xue et al., 2018). At 30-day-old seedling time, statistical analysis of the number of main branches in Transgenic plants and WT.

## Results

### Identification of the TCP Gene Family in Blueberry

All prospective *VcTCP* members were obtained from the blueberry genome using HMMER and BlastP. The domain integrity of the *VcTCP* candidate genes was identified using the SMART database (Supplementary Figure 1). A total of 62 TCP gene family members were identified on 30 chromosomes and named *VcTCP1*-*VcTCP62* according to the order of the chromosomes on which they were located. The TCP domains of some Class II TCP members also included an R domain, which was found at the C-terminus of all VcTCP proteins from blueberry Class II CYC/TB1 and two members of VcTCPs from CIN. Using the online tool ExPASy website, the physical and chemical properties of the VcTCP proteins were analyzed (Table 3). The length of VcTCP proteins varies from 198 (*VcTCP29/47*) to 686 amino acids (*VcTCP24*). *VcTCP47* has the lowest relative molecular mass (20.97 kDa) and the highest is *VcTCP24* (76.58 kDa). Theoretical isoelectric points (pIs) range from 5.2 to 10.02. Aliphatic amino acid indexes are between 51.67 and 78.45, which indicates that the VcTCP proteins are rich in aliphatic amino acids. The average hydrophilicity coefficient of all VcTCP proteins is less than 0, which indicates that VcTCPs are hydrophilic proteins. According to the prediction of WoLF PSORT, most of the VcTCP proteins are located in the nucleus, with only VcTCP26 possibly located in chloroplasts.

**TABLE 3:** Basic information of the TCP gene family in blueberry.

Gene Name	Accession number	Length of amino acid	Chromosome	Chr start	Chr end	MW(Da)	Isoelectric point	Aliphatic index	GRAVY	Subcellular location
*VcTCP1*	VaccDscaff2-snap-gene-363.30	521	2	36372942	36374793	55282.05	8.69	55.43	−0.666	Nucleus
*VcTCP2*	VaccDscaff3-snap-gene-43.34	518	3	4369247	4371162	55022.87	8.69	56.87	−0.642	Nucleus
*VcTCP3*	VaccDscaff4-processed-gene-224.7	271	4	22479281	22480096	30520.6	6.34	62.25	−0.818	Nucleus
*VcTCP4*	VaccDscaff4-processed-gene-248.11	264	4	24835402	24836196	27988.98	9.71	66.33	−0.548	Nucleus
*VcTCP5*	VaccDscaff4-augustus-gene-340.19	328	4	34030814	34032547	36796.64	8.27	55.61	−1.025	Nucleus
*VcTCP6*	VaccDscaff4-processed-gene-349.4	370	4	34921314	34922426	41276.08	9.13	58.24	−0.656	Nucleus
*VcTCP7*	VaccDscaff6-processed-gene-148.0	295	6	14799921	14800808	32062.43	7.24	57.22	−0.724	Nucleus
*VcTCP8*	VaccDscaff6-processed-gene-185.5	342	6	18562851	18563879	36003.7	6.3	51.67	−0.688	Nucleus
*VcTCP9*	VaccDscaff7-snap-gene-330.28	276	7	33039338	33040490	30758.2	9.98	75.33	−0.562	Nucleus
*VcTCP10*	VaccDscaff7-processed-gene-346.11	343	7	34666303	34667334	38297.32	7.14	67.87	−0.799	Nucleus
*VcTCP11*	VaccDscaff7-processed-gene-411.5	308	7	41170553	41171479	32829.45	7.98	68.12	−0.603	Nucleus
*VcTCP12*	VaccDscaff9-processed-gene-173.3	271	9	17311444	17312259	30515.59	6.7	63.32	−0.841	Nucleus
*VcTCP13*	VaccDscaff9-processed-gene-209.15	267	9	20954383	20955186	28245.25	9.71	66.33	−0.542	Nucleus
*VcTCP14*	VaccDscaff9-processed-gene-315.5	328	9	31562489	31564124	36890.75	8.27	55.3	−1.046	Nucleus
*VcTCP15*	VaccDscaff9-processed-gene-325.5	370	9	32506674	32507786	41210.96	9.13	57.97	−0.671	Nucleus
*VcTCP16*	VaccDscaff13-processed-gene-51.0	277	13	5097023	5097856	29409.67	9.3	61.77	−0.646	Nucleus
*VcTCP17*	VaccDscaff13-processed-gene-86.0	332	13	8593748	8594746	37184.47	7.85	71.27	−0.689	Nucleus
*VcTCP18*	VaccDscaff13-augustus-gene-211.25	358	13	21133293	21135461	40445.96	9.22	66.51	−0.893	Nucleus
*VcTCP19*	VaccDscaff13-processed-gene-258.2	390	13	25835319	25837106	41331.25	7.95	56.67	−0.649	Nucleus
*VcTCP20*	VaccDscaff14-snap-gene-312.22	523	14	31200940	31203184	55493.27	8.69	55.22	−0.666	Nucleus
*VcTCP21*	VaccDscaff15-processed-gene-260.4	444	15	26063256	26064590	48913.59	8.67	56.89	−0.968	Nucleus
*VcTCP22*	VaccDscaff16-processed-gene-79.3	343	16	7915380	7916411	38297.32	7.14	67.87	−0.799	Nucleus
*VcTCP23*	VaccDscaff16-snap-gene-95.39	296	16	9488483	9489602	33053.77	10.02	78.45	−0.474	Nucleus
*VcTCP24*	VaccDscaff18-processed-gene-313.1	686	18	31354637	31355668	76576.62	7.32	67.87	−0.799	Nucleus
*VcTCP25*	VaccDscaff19-processed-gene-121.8	365	19	12060048	12095062	41160.03	9.28	59.07	−0.914	Nucleus
*VcTCP26*	VaccDscaff20-snap-gene-23.40	552	20	2370317	2371450	59152.97	7.13	72.25	−0.369	Chloroplast. Nucleus
*VcTCP27*	VaccDscaff20-processed-gene-118.24	354	20	11882803	11887091	39906.4	9.31	53.47	−1.033	Nucleus
*VcTCP28*	VaccDscaff21-processed-gene-72.8	310	21	7278313	7279245	32514.59	5.28	73.06	−0.329	Nucleus
*VcTCP29*	VaccDscaff21-processed-gene-227.4	198	21	22728228	22728824	21039.9	8.03	74.55	−0.269	Nucleus
*VcTCP30*	VaccDscaff24-processed-gene-124.3	444	24	12422046	12423380	48882.62	9.02	57.32	−0.952	Nucleus
*VcTCP31*	VaccDscaff25-snap-gene-361.38	528	25	36090924	36093034	55879.72	8.69	55.62	−0.649	Nucleus
*VcTCP32*	VaccDscaff26-processed-gene-71.5	355	26	7140969	7143147	37502.98	5.2	68.51	−0.407	Nucleus
*VcTCP33*	VaccDscaff28-processed-gene-228.2	372	28	22858134	22859252	41854.53	9.01	54.84	−0.947	Nucleus
*VcTCP34*	VaccDscaff28-processed-gene-231.13	368	28	23127185	23130381	41617.57	8.75	61.49	−0.846	Nucleus
*VcTCP35*	VaccDscaff28-snap-gene-333.33	325	28	33342992	33345897	34246.78	5.71	69.63	−0.303	Nucleus
*VcTCP36*	VaccDscaff29-processed-gene-73.2	378	29	7308827	7311058	40123.07	5.72	70.24	−0.354	Nucleus
*VcTCP37*	VaccDscaff29-processed-gene-216.4	199	29	21606892	21607491	21050.96	7.11	77.14	−0.196	Nucleus
*VcTCP38*	VaccDscaff30-snap-gene-144.30	386	30	14423911	14425903	40926.99	8.65	57.98	−0.658	Nucleus
*VcTCP39*	VaccDscaff30-augustus-gene-188.14	389	30	18850412	18852920	44214.37	9.35	64.99	−0.927	Nucleus
*VcTCP40*	VaccDscaff30-processed-gene-307.4	277	30	30705372	30706205	29409.67	9.3	61.77	−0.646	Nucleus
*VcTCP41*	VaccDscaff30-processed-gene-322.0	333	30	32192637	32193638	37610.96	7.86	68.98	−0.735	Nucleus
*VcTCP42*	VaccDscaff31-snap-gene-5.36	278	31	503433	504550	31042.52	9.98	74.78	−0.584	Nucleus
*VcTCP43*	VaccDscaff32-processed-gene-119.6	380	32	11944947	11946089	40157.08	6.9	58.89	−0.623	Nucleus
*VcTCP44*	VaccDscaff32-augustus-gene-169.22	481	32	16967184	16969298	53571.08	8.44	73.64	−0.676	Nucleus
*VcTCP45*	VaccDscaff32-processed-gene-279.5	334	32	27905053	27906057	37459.86	7.76	69.67	−0.701	Nucleus
*VcTCP46*	VaccDscaff32-snap-gene-314.26	364	32	31384394	31385573	38996.67	9.21	69.75	−0.481	Nucleus
*VcTCP47*	VaccDscaff33-processed-gene-128.0	198	33	12838946	12839542	20965.85	7.11	76.52	−0.218	Nucleus
*VcTCP48*	VaccDscaff33-snap-gene-264.40	376	33	26478805	26481217	40407.54	5.73	72.69	−0.395	Nucleus
*VcTCP49*	VaccDscaff35-processed-gene-150.6	264	35	15016801	15017595	27888.91	9.71	66.7	−0.516	Nucleus
*VcTCP50*	VaccDscaff35-augustus-gene-247.34	328	35	24765835	24767672	36908.77	7.73	55.3	−1.045	Nucleus
*VcTCP51*	VaccDscaff35-processed-gene-256.6	370	35	25680771	25681883	41251.07	9.13	59.3	−0.652	Nucleus
*VcTCP52*	VaccDscaff36-processed-gene-93.1	370	36	9319351	9320463	41214.01	9.13	58.78	−0.66	Nucleus
*VcTCP53*	VaccDscaff36-augustus-gene-102.19	328	36	10282584	10284514	36812.68	8.27	56.8	−1.007	Nucleus
*VcTCP54*	VaccDscaff36-processed-gene-208.0	263	36	20792875	20793666	27960.93	9.71	65.82	−0.577	Nucleus
*VcTCP55*	VaccDscaff37-processed-gene-63.7	294	37	6343639	6344523	32101.53	6.89	58.74	−0.726	Nucleus
*VcTCP56*	VaccDscaff37-processed-gene-97.9	358	37	9772501	9773577	37504.42	6.34	52.68	−0.645	Nucleus
*VcTCP57*	VaccDscaff38-processed-gene-183.2	359	38	18300835	18301914	37561.47	6.34	52.53	−0.645	Nucleus
*VcTCP58*	VaccDscaff38-processed-gene-217.5	294	38	21761403	21762287	32160.64	7.24	58.74	−0.714	Nucleus
*VcTCP59*	VaccDscaff39-processed-gene-243.3	293	39	24323818	24324699	31978.36	7.24	57.61	−0.728	Nucleus
*VcTCP60*	VaccDscaff42-processed-gene-10.1	277	42	1047100	1048163	29409.67	9.3	64.22	−0.646	Nucleus
*VcTCP61*	VaccDscaff42-processed-gene-170.0	379	42	17002895	17004034	40000.9	6.9	59.05	−0.615	Nucleus
*VcTCP62*	VaccDscaff48-augustus-gene-92.26	327	48	9269735	9272947	34578.23	5.83	72.2	−0.287	Nucleus

### Phylogenetic Analysis of the VcTCP Gene Family

**FIGURE 1:**
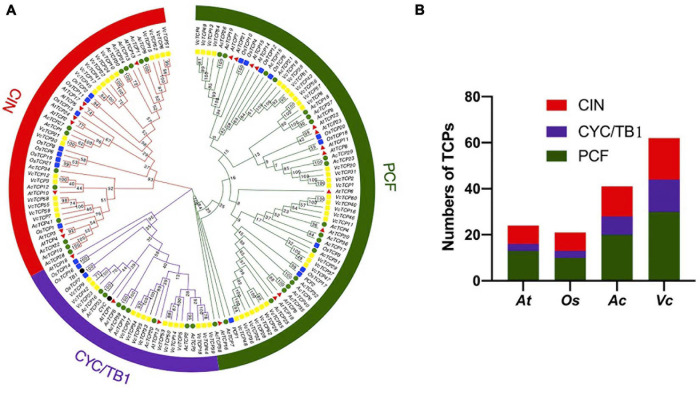
**(A)** Phylogenetic analysis of TCP members in blueberry (*VcTCP* rectangle, yellow), kiwifruit (*AcTCP* circle, green), *Arabidopsis* (*AtTCP* triangle, red), rice (*OsTCP* rectangle, blue), *Antirrhinum CYC* and maize *TB1*(circle, black). The phylogenetic tree was constructed using the neighbor-joining method with 1,000 bootstrap replicates in MEGAX. The branched lines of the subtrees are colored to indicate different TCP subgroups. The number on the branch denotes the corresponding bootstrap value. **(B)** Statistical analysis of TCP members from blueberry, rice, kiwifruit and *Arabidopsis*.

**FIGURE 2:**
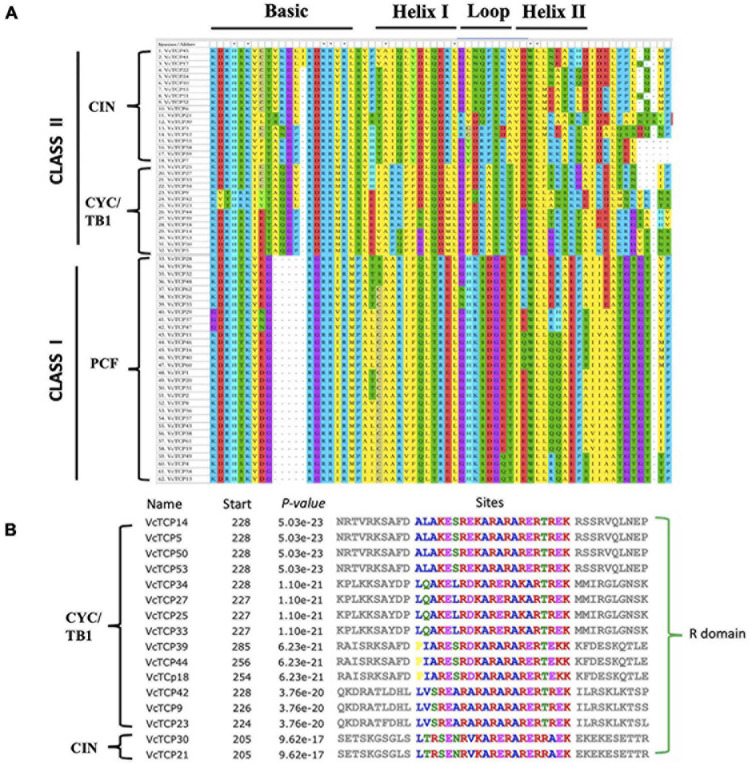
Conserved structural domain analysis of VcTCPs in blueberry. **(A)** Multiple alignment of VcTCPs protein sequences was performed using MEGA X software. **(B)** Multiple alignment of the R domain sequences.

To study the evolutionary and phylogenetic relationships between the blueberry *TCP* genes and other known *TCPs*, the full-length sequences of 150 TCP proteins from *Arabidopsis*, rice, kiwifruit, blueberry, snapdragon CYC and maize TB1 were used to construct a phylogenetic tree. According to previous studies of the classification of *TCP* genes in *Arabidopsis* and rice, phylogenetic analysis and TCP domain alignment all showed that VcTCP proteins can be divided into two categories: class I (PCF), which contains 30 genes; and class II, which contains 32 genes (Figure 1). The phylogenetic tree indicates that class II may be further divided into two subclades, CYC/TB1 (14) and CIN (18). The TCPs of *Arabidopsis* and rice had the same groups or branches, indicating the reliability of the phylogenetic tree. Class II proteins have four more amino acids in the basic domain than class I proteins, which is the most significant difference between these two classes (Figure 2A). The TCP domains of some Class II TCP members also included an R domain, which was found at the C-terminus of all VcTCP proteins from blueberry Class II CYC/TB1 and two members of VcTCPs from CIN (Figure 2B).

### Conserved Motif and Gene Structure Analysis

**FIGURE 3:**
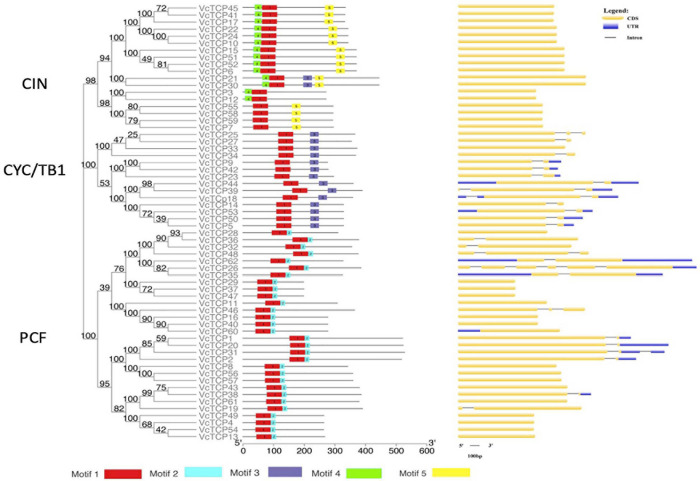
Phylogenetic analysis, conserved motifs and gene structure of the TCP family in blueberry. Conserved motifs of VcTCPs were identified using MEME. Different motifs are shown by different colors. CDS, introns and UTR are indicated by yellow rectangles, black lines and blue rectangles, respectively.

To better understand the diversity of the motif composition of VcTCPs, the MEME online tool was used to predict the conserved motifs of the blueberry TCP gene family. A total of five conserved motifs were identified (Figure 3). The results showed that motif 1 existed in all VcTCP proteins. Motif 2 is located in the C-terminal TCP domain of Class I members with high specificity. Motif 4, 5 exist in members of the CIN subfamily, but it was not observed in the TB1 subfamily. By comparison, all class II TB1 proteins are characterized by motif 3 (R domain), two of the CIN subfamily members *VcTCP21*, *VcTCP30*, which are clustered with *AtTCP2*,*24* also contain an R domain, which is consistent with previous research that the presence of certain motifs in a particular subgroup may play a specific role in these genes (Wei et al., 2016).

To investigate the evolutionary relationship and structural characteristics of VcTCP proteins, an evolutionary tree was constructed using the conserved domain sequences of VcTCPs, which were divided into three subfamilies (Figure 3). Almost all *VcTCP* genes exhibit a highly conserved exon-intron structure. Twenty-six of the 62 *VcTCP* genes have introns, 19 *VcTCP* genes have only one intron, and 7 *VcTCP* genes have two or more introns. Most of the *VcTCP* genes in the same subfamily show similar exon/intron distribution patterns, which supports the classification and evolutionary relationship of the subclade.

### Putative Promoter *Cis-*Element Analysis of Blueberry *VcTCP* Genes

**FIGURE 4:**
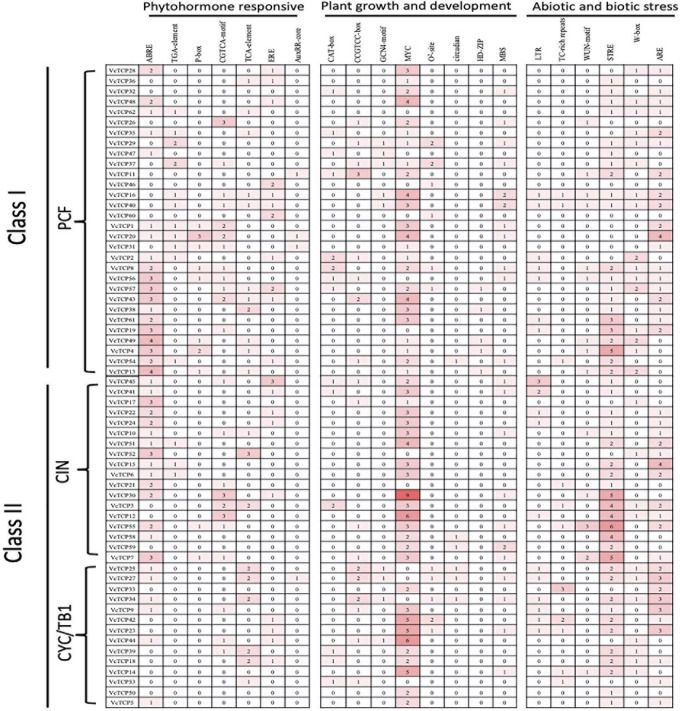
*Cis-*regulatory elements analysis of the promoter region of *VcTCP* genes. Numbers of each *cis-*regulatory element in the promoter region of *VcTCP* genes. Based on the functional annotation, the *cis-*acting elements were classified into three major classes: phytohormone responsive, plant growth and development, and abiotic and biotic stresses.

To better understand the response and regulation mechanisms of *VcTCP* genes, promoter *cis-*regulatory elements of the 62 *VcTCP* genes were analyzed. The sequences of 1,500 bp upstream of the genes were submitted to Plant CARE. In addition to the basic TATA and CAAT boxes, the promoters contain primarily light response, transcription factor binding (MYB and MYC, etc.), hormone response, injury response, low temperature response, zeatin metabolism, flavonoid metabolism regulation and other *cis-*acting elements. Among the 62 *VcTCP* gene promoters, light-responsive (TCT-motif, Sp1, GT1-motif), MYB-binding and MYC-binding elements predominate, and all *VcTCP* genes contain these three types of response elements. Hormone-response elements primarily include abscisic acid (ABRE), methyl jasmonate (CGTCA-motif, TGACG-motif), ethylene (ERE), auxin (TGA-element), salicylic acid (TCA-element, SARE), and gibberellin (P-box). Abscisic acid-responsive elements are the most common among the hormone-response elements, which suggests that abscisic acid regulates this family of transcription factors during growth and development. For the abiotic stress response elements, a large number of low temperature responsiveness (LTR), anaerobic induction (ARE), and drought induction (MBS) elements were detected. These results revealed that *VcTCP* genes play an important role in the response to biotic and abiotic stresses. Some *cis-*regulatory elements of growth and development, hormone response and biological abiotic stress in the present study are depicted in Figure 4.

### Gene Duplication and Synteny Block of *VcTCP* Genes

Gene replication and differentiation are important processes for gene family expansion and novel functional evolution in the plant genome. Some gene replication events, including tandem replication, WGD (Whole Genome Duplication)/segment duplication, and chromosomal and gene-level rearrangements, have driven the evolution of protein-coding gene families (Maher et al., 2006). To evaluate expansion of the VcTCP gene family, we used MCScanX software to analyze the origin of the VcTCP gene family repeat genes in the blueberry genome. In the gene type analysis, *VcTCP58* exist in tandem, the other members derived from WGD/segment duplication (Supplementary Material Additional file 1) which demonstrated that the WGD/segment played an important role in the expansion of the VcTCP gene family.

**FIGURE 5:**
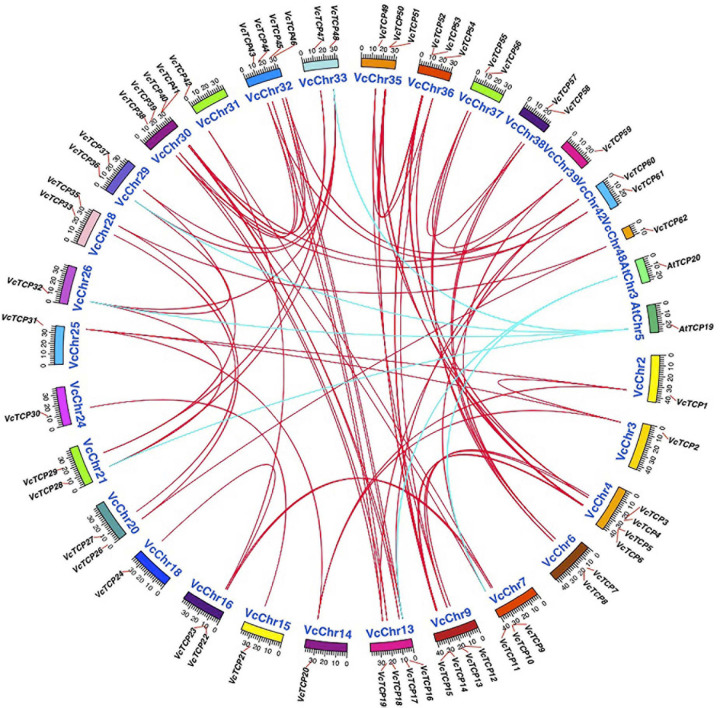
Chromosome distribution and synteny analysis of blueberry and *A. thaliana TCP* genes. Chromosomes of *V. corymbosum* and *A. thaliana* are shown in different colors and circular form. The approximate distribution of *VcTCPs* and *AtTCPs* are marked with a short red line on the circle. Red and blue curves denote the details of syntenic regions between blueberry and *A. thaliana TCP* genes.

To further examine the origin and possible evolutionary mechanism of the VcTCP gene family, the BlastP resulting files of blueberry with *Arabidopsis* and blueberry itself were entered into MCScanX, and the homology matching file was examined. The source pairings were very complicated, and no homologs were found between two genes *VcTCP25* and *VcTCP34*. All other gene family members have collinear relationships, as shown by the red line (Figure 5). Homology analysis showed six homologous gene pairs between blueberry and *Arabidopsis*, as indicated by the blue line. The homology analysis of blueberry and *Arabidopsis* sequences demonstrated that these genes located in the corresponding homology region arose before the divergence of blueberry and *Arabidopsis*.

### Transcript Profiling of *VcTCP* Genes in Tissues, Fruit Development, Bud Dormancy Release, and in Response to Hormone in Blueberry

**FIGURE 6:**
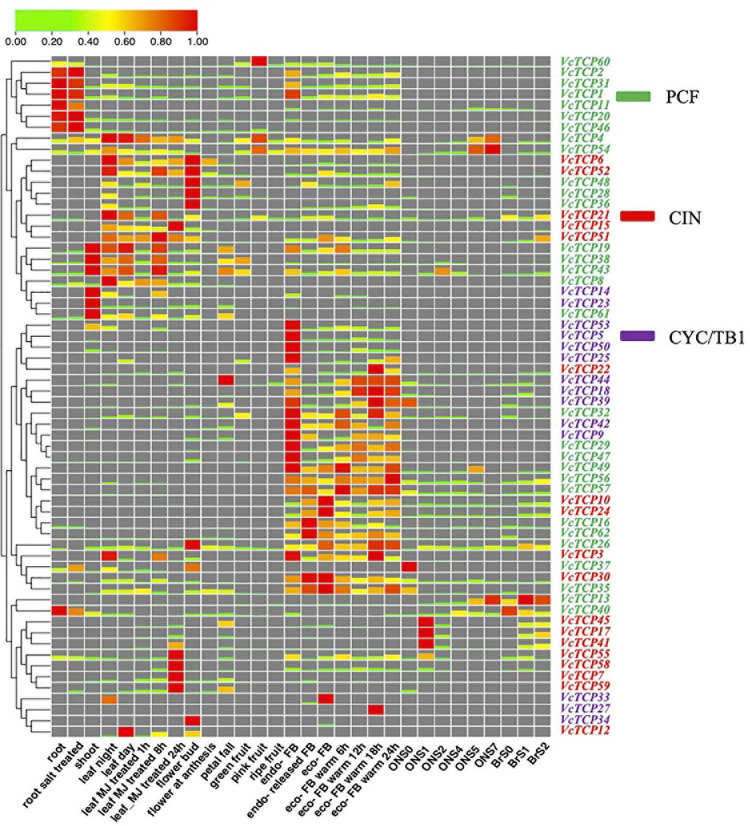
Expression profiles of *VcTCP* genes in diverse tissues, stages of fruit development, flower bud dormancy release, and in response to hormone treatment. The mean expression values were calculated using zero to one. Genes and expression patterns were hierarchically clustered based on the average Pearson’s metric. Green and red boxes show low and high expression levels, respectively, for every gene. Different colors of gene name belong to class I, CIN, and CYC/TB1, respectively. BrS0, S1, and S2 indicated the early fruit developmental stages of the blueberry variety “Blue rain”; ON S0–S7 indicated the whole fruit development of the blueberry variety ‘O'Neal.’ FB means flower bud. Leaf MJ-treatment: leaf methyl jasmonate (MeJA) treatment. Endo- means endo-dormancy; eco- means eco-dormancy.

The expression patterns of *TCP* genes were examined in different tissues, bud dormancy release, and fruit development in blueberry. As indicated in Figure 6, transcriptome profiling showed that the expression patterns of VcTCPs within the same subclade were not similar, which suggests that these genes play different roles in biological processes. The nine members of the class II subclades CYC/TB1 (*VcTCP9/42/23/44/39/14/53/50/5*) exhibited a certain level of expression in the shoot and leaf-night, and these genes were not expressed during fruit development. Before and after root salt treatment, members of this subclade were not expressed. After MeJA treatment, *VcTCP23* was downregulated at 1 and 8 h and upregulated at 24 h. All members of the subclade decreased with cold, but the expression levels increased during the process of eco-dormancy release. During bud endo-dormancy release, the expression levels of the *BRC1* homologous genes *VcTCP5, VcTCP14, VcTCP18, VcTCP39, VcTCP44, VcTCP50*, and *VcTCP53* were obviously decreased; however, the change of expression levels of the *BRC2* (*AtTCP12*) homologous genes *VcTCP25, VcTCP27, VcTCP33*, and *VcTCP34* was not obvious, which is consistent with the results in the literature (Aguilar-Martínez et al., 2007).

Among the CIN members, the transcript levels of *VcTCP21* and *VcTCP30* were high in the root, leaf, flower, and fruit tissues, but the other members were not expressed in the root. The expression levels of *VcTCP21* and *VcTCP30* also exhibited significant differences during fruit development, which indicates that these genes may play a necessary role in the growth and development of root and fruit. The expression levels of *VcTCP41*, *VcTCP17*, *VcTCP58*, *VcTCP59* and *VcTCP7* were increased after MeJA treatment 24 h after treatment, and the transcript accumulation levels exhibited significant differences, which suggests that these genes play a significant role in the JA signal transduction pathway.

Most PCF members were expressed in all tissues. The expression levels were higher in root, shoot, leaf and bud. *VcTCP31*, *VcTCP2*, *VcTCP8*, *VcTCP43*, and *VcTCP38* were downregulated during fruit development. *VcTCP60*, *VcTCP4*, and *VcTCP54* were upregulated in pink fruit and downregulated in ripe fruit, which revealed that these genes participate in blueberry fruit development. The expression levels of *VcTCP43*, *VcTCP38*, *VcTCP61*, *VcTCP19*, and *VcTCP49* decreased 1 h after MeJA treatment, increased after 8 h, and decreased after 24 h. This expression trend was consistent, whether this type of genes plays a specific role in JA signal transduction is not known.

### Expression Patterns of *VcTCP* Genes in Different Tissues and During Flower Bud Endo-Dormancy Release by Artificial Cold Treatment

To elucidate the roles of *TCP* genes in flower bud dormancy release, several *VcTCP* genes were selected for quantitative verification: *VcTCP5*, *VcTCP18*, *VcTCP19*, *VcTCP21*, *VcTCP26*, *VcTCP54*, *VcTCP55*, and *VcTCP60*. These genes belong to the CIN, TB1, and PCF subclasses and show different expression patterns during flower bud dormancy release, fruit development and hormone treatment in transcriptome profiling analysis.

**FIGURE 7:**
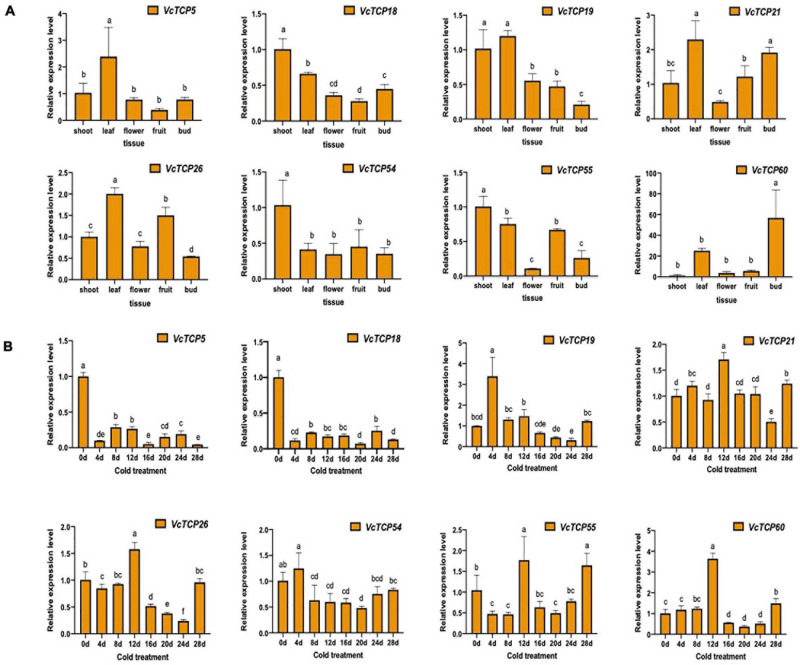
**(A)** Relative expression levels of *VcTCPs* in different tissues of ‘O'Neal’ blueberry. **(B)** Relative expression levels of *VcTCPs* during flower bud endodormancy release by artificial chilling treatment. Values were normalized against the expression data of *VcGAPDH* and given as the means ± standard error of three biological replicates. Different letters indicate significant differences between genes (*p* < *0.05*) based on Duncan test. The expression levels were calculated based on the 2^− ΔΔCt^ method.

To investigate the tissue-specific profiles of *VcTCP* genes in blueberry, we analyzed the expression levels of selected *VcTCP* genes using RT-qPCR to validate the results of transcriptomes of different tissues, including shoots, leaves, flower and fruits from the blueberry accession ‘O'Neal’. Eight *VcTCP* genes exhibited tissue-specific transcript accumulation patterns, indicating the functional divergence of *VcTCP* genes during blueberry growth and development (Figure 7A). For example, the expression levels of *VcTCP5*, *VcTCP19*, and *VcTCP26* were very high in leaves, and there are significant differences compared with other tissues, indicating that these genes might play an important role in leaf development. *VcTCP21* and *VcTCP60* were highly expressed not only in the leaves but also in the buds, which indicated that these genes might play an important role in floral buds. All of these results were consistent with those in the transcriptome.

**FIGURE 8:**
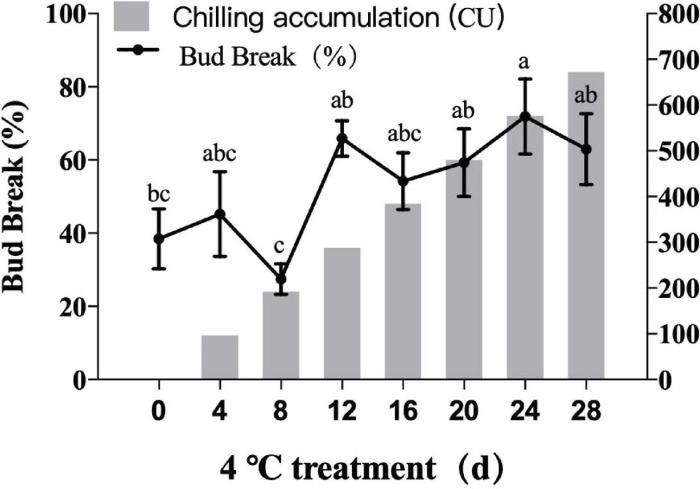
Germination rates of blueberry flower bud under different cold accumulation. All were the highest germination rates.

As illustrated in Figure 8, average germination rates of ‘O'Neal’ flower bud increased with longer cold treatment times. The flower buds were in deep endo-dormancy stage before transfer to 4°C, and the germination rate was low. The sprouting rate of flower buds reached 65.89% after 12 days of cold treatment, which indicated the endodormancy was broken. After that point, flower buds have strong sprouting potential, then enter the eco-dormancy phase.

As shown in Figure 7B, *VcTCP18* and *VcTCP5* belong to the TB1 subclass and were highly expressed during the deep dormancy period (0 d); their expression levels decreased significantly when the dormant flower buds were subjected to 4 days of cold treatment. The expression of the two genes remained at a consistently low level with increasing duration of the cold treatment, which indicates that the two genes may inhibit bud germination. The expression levels were reduced after cold treatment, which restored the ability of the flower bud to restore germination.

*VcTCP21* and *VcTCP55* belong to the CIN subcategory, and their expression levels were increased significantly after 12 days of cold treatment. Notably, the germination of blueberry flower buds over 50% at this stage indicates that the genes may participate in flower bud endo-dormancy release. *VcTCP26*, *VcTCP54*, *VcTCP19*, and *VcTCP60* belong to the PCF subclass, and the expression patterns in the cold treatment increased, decreased, then increased. The highest expression levels of *VcTCP26* and *VcTCP60* were observed after 12 days of cold treatment, but their expression levels increased a second time after cold treatment for 28 days. The flower buds were in the eco-dormancy stage at this time and germinate quickly if given the appropriate temperature. The expression patterns of *VcTCP54* and *VcTCP19* were the same as described above but occurred after 4 days of treatment, which indicates that these genes also responded to low temperature.

### Clone and Genetic Transformation of *VcTCP18*

*BRC1* has been shown to be the central integrator for multiple environmental and developmental factors that functions locally to inhibit bud break. Previous research has shown that *DAM* can be regulated by low temperature, which play a negative regulatory role in bud break, and *TCP18/BRC1* is a downstream target gene of *SVL/DAM* (Singh et al., 2018). These observations prompted us to investigate *BRC1*s role in the blueberries bud dormancy release.

According to the analysis of gene expression in Figure 7B and the phylogenetic tree of CYC/TB1 subclade, shown in Supplementary Figure 2, although there are other putative *BRC1-like* genes in blueberry, *VcTCP18* was selected in the present study, due to its profile that fit with an expected putative *BRC1* gene for further analysis. A cDNA fragment with a length of 1,077 bp was obtained, as shown in Supplementary Figure 3A. The coding sequences were cloned into the vector pCAMBIA2300. Finally, the recombinant plasmid vector was transferred into *Agrobacterium* strain GV3101.

### Phenotypes of Transgenic *Arabidopsis* With Overexpression of *VcTCP18*

**FIGURE 9:**
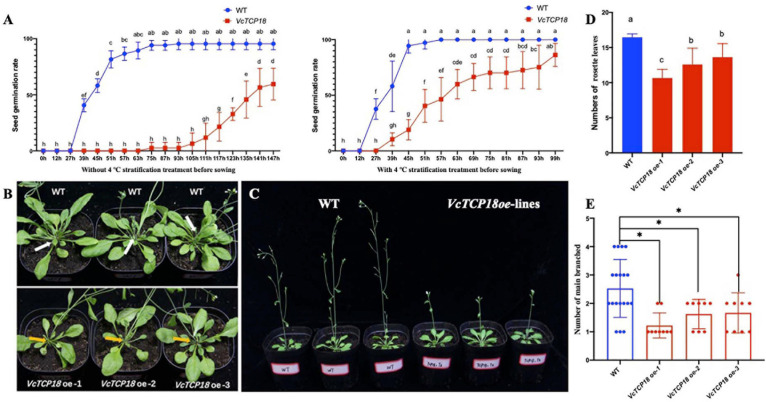
Phenotypes of transgenic *Arabidopsis* with overexpression of *VcTCP18*. **(A)** The seed germination rate of WT and transgenic lines with and without 4°C stratification treatment before sowing. **(B)** 30-day-old seedling observation of main branches, white and yellow arrows indicate main branches of WT and transgenic lines, respectively. **(C)** Flowering time observation, three lines on left are WT, the others on right are *VcTCP18*-overexpressed transgenic lines. **(D)** At first flowering time, the numbers of rosette leaves, different letters indicate significant differences between WT and transgenic lines (*p* < *0.05*), statistical analysis was performed by one-way ANOVA followed by the Duncan test. **(E)** statistical analysis of the number of main branches in Transgenic plants and WT, each dot represents one plant. Asterisks (^∗^) indicate significant differences (*P* < *0.05*) based on Duncan test.

Through the Kana screening test, we obtained the T1 generation *Arabidopsis* transgenic seedlings. The further RT-PCR experiments and sequencing results showed that *VcTCP18* was successfully expressed in *Arabidopsis* (Supplementary Figure 3B). Then the germination of T3 homozygous transgenic seeds were tested on 1/2 MS with and without stratification (Figure 9A). Without stratification treatment, the germination rate of transgenic seeds was much lower than that of WT. the germination rate of WT was first beyond 90% at 75 h after sowing, meanwhile, that of transgenic seeds was 2.5%, the rate of germination of WT was 59.48% until 147 h after sowing. Comparatively, when the seeds were treated by 4°C for 2 days before sowing, both of the seeds of WT and transgenic lines germinated faster than without stratification, however, transgenic seeds still germinated significantly slower than that of WT.

When flower buds were visible by the naked eye, the numbers of rosette leaves were counted (Figures 9B,D). The rosette leaves of WT were 16.51, which significantly differed from *VcTCP18* overexpression lines (*P* < *0.05*). The T3 generation of *VcTCP18* overexpressed plants displayed the late-flowering phenotype (Figure 9C). At 30-day-old seedlings time, the number of main branches in transgenic plants was lower than WT (Figures 9B,E). In the three transgenic lines, with the increase of expression efficiency of *VcTCP18* (Supplementary Figure 4), the number of rosette leaves and main branches decreased, which significantly differed from that of WT. These *VcTCP18*−overexpressing lines exhibited bud break compared with the wild-type.

## Discussion

### TCP Gene Family in Blueberry and Their Evolution

TCPs are a type of plant-specific transcription factor family. Previous studies have used the model plants *Arabidopsis* and rice as research objects to analyze the various functions of TCPs in plant growth and development. The present study identified 62 TCP gene family members in blueberry, compared with *Arabidopsis* (24 members), strawberry (19 members), tomato (20 members), apple (52 members), there were more numbers of *TCP* genes in blueberry. One possible reason is that whole-chromosome sequencing was performed, and blueberry has a large genome (1.63 GB). Another possible reason is that the blueberry variety ‘Draper’ is tetraploid, and thus gene replication events occurred during the evolution of the *VcTCP* genes. Gene replication and amplification events are widespread in the evolution of many plants, such as cotton (Ma et al., 2014), tomato (Parapunova et al., 2014), and apple (Xu et al., 2014).

Analysis of the structure of *VcTCP* genes showed that the genes in the same subclass had a similar gene structure, though the length of the *VcTCP* genes is quite different, which indicates that the *VcTCP* genes are evolutionarily conserved. There are also some *VcTCP* gene structures that are identical, but the other members are obviously different. This phenomenon has also been observed in the structural analysis of TCP gene family members in plants such as strawberry (Wei et al., 2016), cotton (Ma et al., 2014) and apple (Xu et al., 2014). These differences may be caused by the insertion or loss of introns during the evolution of the species, which indicates that the diverse functions and expression control methods of the TCP gene family have involved more replication fragments and gene doubling events during evolution. In our study, we found an arginine-rich R domain in the C-terminus of all VcTCP proteins from blueberry Class II CYC/TB1 and two members of VcTCPs (VcTCP21, VcTCP30) from CIN, which was predicted to promote protein interactions, as is the case for four genes in rice (*OsTCP6*, *OsTCP7*, *OsTCP13*, and *OsTCP14*), four genes (*AtTCP2*, *AtTCP12*, *AtTCP18*, and *AtTCP24*) in *Arabidopsis*, and maize TB1. Our result was concordant with previous studies in grape and tomato (Parapunova et al., 2014; Leng et al., 2019).

When plants suffer from environmental stresses, such as low temperature, drought, salinity and so on, these stresses trigger signaling cascades that activate transcriptional factors, by binding to specific *cis-*acting elements to regulate the expression of genes. The result showed that among 62 *VcTCP* promoters all contain light-responsive (TCT-motif, Sp1, and GT1-motif), MYB-binding and MYC-binding elements. *MYB*, *MYC* participates in drought, low temperature, salt, ABA, and GA stress responses (Griffiths et al., 2006), which indicate *TCP* genes appeared to be regulated by MYB and MYC. For the abiotic stress response elements, a large number of low temperature responsiveness (LTR), anaerobic induction (ARE), and drought induction (MBS) elements also were detected. These results revealed that *VcTCP* genes play an important role in the response to biotic and abiotic stresses.

Previous studies found that the expansion of gene families is mainly caused by gene duplication events, including WGD, segmental duplication, and tandem duplication, and so on (Maher et al., 2006). Our results showed that except for *VcTCP58* from the tandem repeat event, the other members derived from WGD/segment duplication, which demonstrated that the WGD/segment duplication is a predominant type in the expansion of the VcTCP gene family in blueberry.

### Roles of *VcTCP18* Gene in the Release of Blueberry Flower Bud Dormancy

Analysis of the evolutionary tree of the homologs showed that *VcTCP5*, *VcTCP14*, *VcTCP18*, *VcTCP39*, *VcTCP44*, *VcTCP50*, and *VcTCP53*, were closely related to *AtTCP18* (*BRC1*). These *TCP* genes encode proteins that may have the same biological functions in bud development. RT-qPCR experiments found that *VcTCP5* and *VcTCP18* had high expression during the stage of depth endodormancy. When the blueberries were subjected to low temperature, the expression levels decreased rapidly. During the process of endo-dormancy release, the expression levels were maintained at a relatively low level, indicating that low temperature affected these genes expression, which may participate in the release of endodormancy of flower buds.

The experiments with *VcTCP18* transgene study in *Arabidopsis* suggested that the germination of transgenic seeds was markedly inhibited, however, the phenomenon can be alleviated to some extent by stratification. Earlier studies have indicated that there are similar mechanisms between seed and bud break (Fu et al., 2014; Wang et al., 2016). For example, the key ABA biosynthesis gene *NCED3* controls normal ABA accumulation in flower buds, which seem to be key in regulating ABA biosynthesis to induce seed and bud dormancy. *CYP707As* control ABA inactivation, leading to dormancy release. A recent study found that BRC1 can bound to and positively regulates the transcription of three related genes, *HB21*, *HB40*, *HB53*, which belong to a transcription factor family that encodes a class of homeodomain leucine zipper protein (HD-ZIP). These genes are necessary and sufficient to enhance *NCED3* expression (González-Grandío et al., 2017); This finding demonstrated the direct relationship between *BRC1* and ABA signaling. Previous studies identified the *SVL* in poplar, which is similar to the *DAM*, and its downstream target gene is just *TCP18*/*BRC1*, indicating that *SVL* regulates *TCP18*/*BRC1* and form a temperature-responsive transcription element to participate in dormancy release. low temperature induces the down expression of *DAM* genes, leading bud and seed dormancy release; however, overexpression of *DAM* genes can delay bud break. The results of seed germination experiments with transgenic *Arabidopsis* indicate that *VcTCP18* is an important inhibitor in the release of seed dormancy, but the results of our study also proved that the *VcTCP18* mediate the dormancy is not a long-term mechanism. If given chilling during the critical period, even the *VcTCP18* gene still have a high expression, but could not alter the dormancy release process. Just as hypothesis of the previous (Singh et al., 2018), low temperature downregulates *TCP18/BRC1* expression and prevents its binding to FT, leading to the normal functioning of *FT*. This positive feedback loop promotes bud germination; however, once the plant rhythm gene *FT* plays its role, it cannot prevent this transformation process. Studies in pears have shown that once endodormancy in the flower bud is lifted, it enters ecological dormancy, although exogenous ABA application cannot reverse this process (Yang et al., 2020).

Previous research has demonstrated that BRC1 physically interacts with FT2, and BRC1-FT interaction triggers growth cessation by antagonizing FT action (Maurya et al., 2020). Some studies also indicated that BRC1 interacted with FT and TSF (TWIN SISTER OF FT) to regulate the activity of florigen in axillary buds and prevented the premature flowering transformation of axillary meristems (Niwa et al., 2013; Li et al., 2019). *BRC1* transcription is auxin responsive, and acts downstream of strigolactone in *Arabidopsis*, *BRC1* determines bud activation potential but is dispensable for bud growth inhibition (Aguilar-Martínez et al., 2007). The mutant plants display an increasing amount of the rosette branches, on the contrary, little lateral branches were observed in over-expressed *AtTCP18 Arabidopsis* plants. The cucumber TCP family gene *CsBRC1* directly inhibits the expression of *CsPIN3*, which promotes auxin accumulation in the axillary buds and inhibits the germination of the axillary buds (Shen et al., 2019). The main branches were observed at 30-day-old seedlings, the *VcTCP18* transgenic plants have only one or two main branches, but there were three to four main branches in WT. The results indicated that *VcTCP18* can inhibit the outgrowth of axillary buds.

By transgenic experiments, we have good reason to believe that *VcTCP18* can negatively regulate the release of flower bud dormancy. In addition to the vegetative growth cycle, woody plants undergo flowering transitions. Flower buds go through dormancy, are released from endodormancy by low temperature accumulation, and eventually germinate. Therefore, the *BRC1* transcription factor plays an important role in flowering transition, dormancy release in perennial plants, and its specific functions must be further studied.

## Conclusion

The present study firstly identified 62 *TCP* genes in the blueberry genome and analyzed their phylogeny, multiple sequence alignments, gene structure, conserved motifs, and homologies. Transcriptome data were used to evaluate the tissue specificity of TCP gene family members, flower bud dormancy release, fruit development stage, and hormone treatment (Me-JA). qRT-PCR was employed to examine the expression patterns of TCP gene family members during the process of flower bud dormancy release at low temperature. the seed germination rate of *VcTCP18* transgenic *Arabidopsis* was lower and later than that of wild type. The bud dormancy phenomena as later flowering, fewer rosettes and main branches were also observed in transgenic plants, which indicates that flower bud dormancy release can be negatively regulated by *VcTCP18* in blueberry. Overall, our study will deepen our understanding of the function of *TCPs* in plant growth and development.

## Data Availability Statement

Publicly available datasets were analyzed in this study. This data can be found here: https://www.vaccinium.org/bio_data/968095 and https://www.vaccinium.org/node/854703.

## Author Contributions

YL: conceptualization, software, writing—original draft preparation, writing—review and editing, visualization, and project administration. YZ: methodology. SA: validation, formal analysis, and data curation. QC: investigation. WG: resources. WC: supervision. YL, LZ, and WG: funding acquisition. All authors have read and agreed to the published version of the manuscript.

## Conflict of Interest

The authors declare that the research was conducted in the absence of any commercial or financial relationships that could be construed as a potential conflict of interest.

## Footnotes

1 http://pfam.janelia.org

2 http://www.arabidopsis.org

3 http://smart.embl-heidelberg.de/

4 https://web.expasy.org/protparam/

5 http://www.genscript.com/psort/wolf_psort.html

6 http://planttfdb.cbi.pku.edu.cn

7 https://www.ncbi.nlm.nih.gov/

8 http://meme-suite.org/tools/meme

9 http://gsds.cbi.pku.edu.cn

10 http://bioinformatics.psb.ugent.be/webtools/plantcare/html/

11 http://circos.ca/

## Supplementary Material

The Supplementary Material for this article can be found online at Figshare via the doi: 10.6084/m9.figshare.14445723.

Supplementary Figure 1 The domain integrity of the *VcTCP* candidate genes was identified using the SMART database.

Supplementary Figure 2 Phylogenetic analysis of Class II CYC/TB1 TCPs in blueberry and AtTCP1, AtTCP12, and AtTCP18. The phylogenetic tree was constructed using the neighbor-joining method with 1,000 bootstrap replicates in MEGAX.

Supplementary Figure 3 Analysis of electrophoretic profiles. **(A)** Bands of *VcTCP18* full-length (1,077 bp) cDNA after gel electrophoresis. **(B)** Semi quantitative RT-PCR assay detected *VcTCP18* products of 86 bp on partial T1 *Arabidopsis* transgenic seedlings.

Supplementary Figure 4 Expression levels of *VcTCP18* in different transgenic plants. **(A)** Semi quantitative RT-PCR assay, *VcGADPH* was used as internal reference. Lane 1, marker; lane 2–4, *internal* reference; lane 5–7, *VcTCP18 oe-1*, *VcTCP18 oe-2, and VcTCP18 oe-3*; bands of *VcTCP18* full-length (1,077 bp). **(B)** Relative expression levels of *VcTCP18* in three transgenic lines. Values were normalized against the expression data of *VcGAPDH* and are given as the means ± SD of three biological replicates. The expression levels were calculated based on the 2^–ΔΔCt^ method.
